# Prevalence and Mutation Analysis of Short-Chain acyl-CoA Dehydrogenase Deficiency Detected by Newborn Screening in Hefei, China

**DOI:** 10.3390/ijns10040068

**Published:** 2024-10-02

**Authors:** Haili Hu, Qingqing Ma, Weidong Li, Yan Wang, Wangsheng Song, Yong Huang

**Affiliations:** 1 Anhui Women and Children’s Medical Center, Hefei 230001, China; topgun_hy@aliyun.com; 2 Department of Children’s Health, Maternal and Child Medical Center, Anhui Medical University, Hefei 230001, China; 3 Hefei Women and Children Health Center, Hefei 230092, China; mqq1099@163.com (Q.M.); gujinw20l@126.com (W.L.); wangyan20091003@126.com (Y.W.); sws8104@163.com (W.S.)

**Keywords:** short-chain acyl CoA dehydrogenase deficiency, newborn screening, tandem mass spectrometry, genetic mutation

## Abstract

Short-chain acyl-CoA dehydrogenase deficiency (SCADD) is an autosomal recessive inborn error of mitochondrial fatty acid oxidation with highly variable biochemical and genetic characteristics. The present study aimed to estimate the prevalence and genetic characteristics of SCADD in newborns identified through screening. A total of 782,930 newborns were screened for SCADD in Hefei Neonatal Screening Center from January 2016 to December 2023. The blood samples from newborns were measured by tandem mass spectrometry (MS/MS). The suspected SCADD neonates were rechecked using next-generation gene sequencing for diagnosis. Sanger sequencing was used to verify the mutation site for patients with SCADD and their parents. A total of 21 SCADD cases were confirmed, with an incidence rate of 1/37,282. Genetic mutations were identified in all 21 cases, including 15 cases of compound heterozygous variation and 6 cases of homozygous variation. Twenty-one different mutation types and forty-two mutation sites were discovered, with the most frequent mutation being c.1031A>G, accounting for 21.43% (9/42), followed by c.1130C>T, accounting for 16.67% (7/42). Our findings expand the SCADD mutational spectra. c. 1031A>G and c.1130C>T are the common mutation sites for SCADD genes in newborns. SCADD diagnosed through NBS is primarily a benign condition, and early diagnosis is not necessarily essential.

## 1. Introduction

The short-chain acyl-CoA dehydrogenase deficiency (SCADD) is a rare autosomal recessive mitochondrial disorder of fatty acid β-oxidation and is associated with mutations in the acyl-CoA dehydrogenase gene (ACADS) [1]. Patients with SCADD present diverse clinical manifestations, such as failure to thrive, hypoglycemia, seizures, hypotonia, mental retardation, and behavioral abnormalities [2]. Research on patients identified through newborn screening (NBS) in Georgia, California, and Illinois has demonstrated that these patients remain asymptomatic and do not exhibit major health problems [3,4,5]. Recent studies have suggested that SCADD may represent a biochemical phenotype with no clear clinical consequences and recommend against treatment with carnitine or riboflavin supplementation [6]. Elevated circulating butyrylcarnitine (C4) levels can serve as a primary marker for the screening and diagnosis of SCADD in infants [7]. In recent years, the widespread adoption of tandem mass spectrometry (MS/MS) in NBS for genetic metabolic diseases has led to an increasing number of SCADD cases being identified in neonates. However, C4 acylcarnitine also increases in other disorders, including multiple acyl-CoA dehydrogenase deficiency (MADD) and isobutyryl-CoA dehydrogenase deficiency (IBDD). In many cases, DNA sequencing is necessary to confirm or rule out true SCADD and to exclude other disorders from the differential diagnosis. The most appropriate next diagnostic test is molecular genetic testing, beginning with sequence analysis of the ACADS gene. Whole exome or genome sequencing may provide additional insights into the pathogenesis and help determine whether other genes could contribute to the disease [8]. Timely diagnosis and treatment can effectively prevent organ damage and mitigate the impact on growth and development in affected children. The present study aimed to investigate the incidence rate of SCADD and the distribution of C4 levels in children in Hefei from 2016 to 2023 through MS/MS. Additionally, genetic testing was conducted to gain insights into the genetic characteristics of children with SCADD in this area and to identify the main types of gene mutations. These findings will provide a theoretical basis and reference data for the screening, diagnosis, and treatment of SCADD.

## 2. Materials and Methods

### 2.1. Study Population

A total of 782,930 newborns delivered at midwifery institutions in Hefei from January 2016 to December 2023 were included in the present study. Written informed consent was obtained from the parents or guardians of all newborns. Our study was approved by the Ethics Committee of Anhui Women and Children’s Medical Center (Approval No: 2016-003; 2024-011), in accordance with the ethical standards of the Helsinki Declaration and its later amendments or comparable ethical standards.

### 2.2. Newborn Screening for SCADD

Heel blood samples were collected after the newborns reached 3 to 7 days of age and were breastfed, and dried blood filter papers were made and transported to the laboratory of Hefei Neonatal Disease Screening Center for testing. The dried blood spot (DBS) samples were prepared using the Panthera-Puncher 9 (PerkinElmer, Turku, Finland) and subsequently analyzed by MS/MS (Xevo TQD, Waters, Milford, MA, USA). The acylcarnitine profiles were determined using underivatized kits produced by PerkinElmer (Turku, Finland) in strict accordance with standard laboratory operating procedures. Non-derivatized MS/MS kits (PerkinElmer, Turku, Finland) were used according to the manufacturer’s guidelines to detect various biochemical indicators, including amino acids and acylcarnitine.

Given the skewed distribution of circulating C4 concentration and the regional differences in reference values for newborn screening, we employed the percentile method to establish the upper and lower limits for the cutoff values [9]. The reference range for C4 in our study was established in 2015 on the basis of neonatal screening data from the Hefei region; the upper and lower limits were determined using the 99.5th and 0.5th percentiles, respectively. Screening conclusions were based on the C4 levels, with a reference range of 0.08 to 0.49 μmol/L. If the initial screening reveals a blood C4 level above 0.49 μmol/L, it is considered a suspected case, necessitating further examination by recalling the newborn for retesting. If the retest continues to show a positive result, both the newborn and their parents are summoned for genetic testing to confirm the diagnosis.

### 2.3. Genetic Analysis

A quantity of 3 mL of whole blood samples were collected from neonates who exhibited abnormal MS/MS testing results as well as their parents. The next-generation sequencing (NGS) platform was used for genetic analysis of the ACADS gene. Following the guidelines of the American College of Medical Genetics and Genomics, the pathogenicity of the identified variants was assessed. Sanger sequencing was performed to validate the positive variants identified by NGS.

### 2.4. Statistical Analysis

The study data were processed using Excel spreadsheets. The disease detection rate was calculated as the ratio of diagnosed cases to the total number of screenings (represented by 1/*n*). Statistical analysis was conducted using SPSS 26.0 (SPSS Inc., Chicago, IL, USA). Data following a normal distribution were presented as the mean ± standard deviation. The t-test was used to compare differences between SCADD and non-SCADD groups, and a significance level of *p* < 0.05 was considered statistically significant (α = 0.05).

## 3. Results

### 3.1. Baseline Characteristics

From 2016 to 2023, a total of 814,022 neonatal live births were documented across all midwifery institutions in Hefei. Among these births, NBS for various genetic metabolic diseases using MS/MS was successfully conducted in 782,930 newborns, achieving a commendable screening rate of 96.18%. Furthermore, the screening rate exhibited consistent annual growth. Among 782,930 participants, 322 were initially screened as positive, of whom 57 remained positive in the recall test. Ultimately, the screenings identified 21 newborns with SCADD using NGS, yielding an overall detection rate of 1 in 37,282 births in Hefei (Table 1). There were 301 false positives, resulting in an overall false-positive rate of 0.04%.

### 3.2. Biochemical Characteristics of Newborns with SCADD

Among the 21 neonates with SCADD, there were 10 boys and 11 girls; 18 were born at term and 3 were born prematurely; 17 had a normal birth weight, 3 had low birth weights, and 1 was macrosomic. None of these neonates exhibited any other significant defects or abnormalities, and no clinical symptoms corresponding to SCADD were observed at the time of diagnosis. Among the neonates diagnosed with SCADD, the average blood C4 level was 1.44 ± 0.46 μmol/L (normal range: 0.08–0.49 μmol/L), with average values of C4/acetylcarnitine (C2), C4/propionyl carnitine (C3), C4/isovalerylcarnitine (C5), and C4/hexanoylcarnitine (C6) of 0.10 ± 0.05 (normal range: 0–0.03), 1.04 ± 0.53 (normal range: 0.04–0.40), 8.90 ± 3.59, and 41.57 ± 15.86, respectively. The average C4 level was 1.33 ± 0.54 μmol/L in male neonates and 1.56 ± 0.34 μmol/L in female neonates, with no statistically significant difference between the two groups (*p* = 0.398). Similarly, premature neonates had an average C4 level of 1.44 ± 0.17 μmol/L, and term neonates had an average C4 level of 1.44 ± 0.50 μmol/L, with no significant difference observed (*p* = 0.936). Furthermore, the average C4 levels in low birth weight, normal birth weight, and macrosomic neonates were 1.44 ± 0.17 μmol/L, 1.44 ± 0.52 μmol/L, and 1.41 μmol/L, respectively, with no statistically significant differences between these groups (*p* = 0.988). In the secondary screening, 12 neonates showed a decrease in blood C4 levels compared with the initial screening, while 9 showed an increase (Table 2).

### 3.3. Genetic Analysis of Newborns with SCADD

All 21 neonates with SCADD were diagnosed with NGS, and the positive variants were confirmed by Sanger sequencing. Among them, 15 cases exhibited compound heterozygous mutations, while 6 cases demonstrated homozygous mutations. Gene mutations were found in multiple exons and introns of the ACADS gene, including exon 2, exon 3, exon 4, exon 5, exon 7, exon 8, exon 9, exon10, intron5, intron6, and intron7. Pathogenic or likely pathogenic mutations were detected in eighteen of the newborns with SCADD. Additionally, a pair of female twins with SCADD were found to carry the genetic variants c.203C>G (p.A68V) and c.373G>C (p.G125R), and one male newborn was found to have the genetic variants c.624+1G>T and c.1210G>A (p.G404R). Although these four mutations were classified as variants of uncertain significance according to the ACMG rating system, our study determined them to be pathogenic variants. In total, 42 mutations in the ACADS gene were identified in the 21 newborns with SCADD. The most prevalent mutation was c.1031A>G, accounting for 21.43% (9/42) of the total. Subsequently, the second most common mutation was c.1130C>T (16.67%, 7/42), and c.1192C>T emerged as the third most frequent mutation, accounting for 7.14% (3/42) of all cases. The c.1054G>A, c.164C>T, c.203C>G, c.373G>C, and c.989G>A mutations each occurred twice. The remaining mutations, including c.1066G>A, c.1156C>T, c.1157G>A, c.1210G>A, c.242C>A, c.320G>A, c.570G>A, c.578C>T, c.624+1G>T, c.795+1G>A, c.815G>A, c.933+1G>A, and c.991G>A, were each detected once (Table 2).

### 3.4. Results of Urinary Ethylmalonic Acid (EMA) Testing

A total of 57 newborns tested positive on the recall test, of whom 24 newborns with positive retests underwent the second-tier test for urine ethylmalonic acid. Among the 24 newborns who underwent the second-tier test, 14 exhibited positive results for ethylmalonic acid in their urine. Among the 21 infants with compound heterozygous or homozygous mutations in the ACADS gene, 13 underwent urinary organic acid testing, which consistently revealed elevated EMA levels in all cases. Of the six infants with compound heterozygous or homozygous mutations in the ACAD8 gene, five received urinary organic acid testing, with four demonstrating increased levels of isobutyrylglycine and one showing no significant abnormalities. Among the five infants carrying ACADS gene variants, four underwent urinary organic acid testing; one exhibited elevated EMA levels, while three showed no significant abnormalities. Of the two infants carrying ACAD8 gene variants, one underwent urinary organic acid testing, which revealed no significant abnormalities.

### 3.5. Clinical Outcomes of Infants with SCADD

We followed 16 infants with SCADD for 16–78 months, while 5 other infants were lost to follow-up. Among the 16 infants with SCADD who were followed up, 2 exhibited clinical symptoms, while the remaining 14 had normal physical, motor, and language development, showing no clinical symptoms. Case 10, a 10-month-old child, presented with fever, vomiting, and diarrhea, which were subsequently followed by convulsions, altered consciousness, hypoglycemia, hypokalemia, and hepatomegaly. Following medical treatment, the symptoms improved. After discharge, a low-fat, high-carbohydrate diet was administered, along with oral supplementation of L-carnitine and riboflavin, while avoiding hunger. Case 19 reported two episodes of febrile seizures, with no other significant symptoms observed. The febrile seizures observed in case 19 may not have been directly caused by SCADD, as a definitive differential diagnosis could not be established at that time.

## 4. Discussion

NBS for genetic metabolic diseases using MS/MS was initiated in Hefei in 2015; the Hefei municipal government began to implement partial financial subsidies for newborn screening in the region as early as 2016, and fully free testing has been implemented since 2018. There has been a consistent rise in the screening rate in recent years, and the NBS rate has reached approximately 99%. The incidence of SCADD in our study population is lower than that found in NBS conducted in various regions of China. For example, Zhao et al. screened 111,547 newborns in Fujian from 2016 to 2022, identifying an incidence rate of 1 in 13,943 for SCADD [10], and 608,818 newborns were screened in Shandong from 2014 to 2019, yielding an incidence rate of 1 in 14,496 [11]. A recent study describing 19-year screening data from Shanghai showed that 81 out of 1,176,073 newborns were diagnosed with fatty acid oxidation disorders, resulting in an incidence rate of 1 in 14,701 [12]. Another study involving 245,194 newborns from 2016 to 2021 in the eastern coastal regions of China revealed that the overall incidence of SCADD was 1 in 30,649 [13]. In addition, the incidence of SCADD identified through NBS was 1 in 15,841 in Hunan [14], 1 in 20,121 in Beijing [15], 1 in 24,384 in Sichuan [16], and 1 in 29,371 in Changsha [17]. Nevertheless, the incidence of SCADD in the present study exceeds the results of NBS conducted in Shijiazhuang, Gansu, and Zhejiang [18,19,20]. However, a study involving 2,632,058 newborns in California from 2005 to 2010 reported an incidence of SCADD of 1 in 34,632 [3], which is relatively close to the findings of our study. There seems to be regional variation in the incidence of SCADD, which may be attributed to regional disparities or differences in sample size. In addition, the incidence of fatty acid oxidation disorders also varies widely between ethnic groups; a combined incidence of 1:9300 newborns was described in reports from Australia, Germany, and the USA [21]. However, it appears to be much lower in Asians, and data from NBS in countries with different ethnic backgrounds reveal significantly different incidences [21]. A molecular genetic study performed on 62 patients in Slovakia found that the presence of sequence variants in the ACADS gene appears with a high frequency in the Roma ethnic group [22].

Infants with SCADD typically exhibit elevated C4 levels as the primary manifestation; 21 neonates diagnosed with SCADD in our study exhibited C4 levels significantly exceeding the normal range. These elevated C4/C2 and C4/C3 ratios further confirm their efficacy as reliable markers for screening newborns with SCADD. C4/C5 and C4/C6 were also considered optimal indicators for distinguishing between different rare diseases that share the same primary biomarker [23]. It should be noted that since genetic testing was not performed on all newborns who participated in the screening, the false-positive rate estimate is based on the premise that C4 diagnosis has a very high specificity of 99.99% [24]. Furthermore, there were no reported cases of missed diagnosis during the eight years of follow-up in the Hefei area. Additionally, Song et al. [25] employed a cut-off value of C5 ≥ 0.44 μmol/L as the diagnostic standard for SCADD, but its sensitivity was only 60%. Our study compared C4 levels among newborns of different genders, birth weights, and gestational ages. The results were all statistically insignificant, indicating that C4 levels in patients with SCADD are not influenced by these factors. However, one of the main challenges of newborn screening programs is to cut down on false positives in order to avoid family stresses regarding newborns and unnecessary costs [26]. The C4 value alone is not enough to guarantee the establishment that the sample is a true or false positive. Post-analytical tools have been developed to reduce false positive results by focusing on the inclusion of multiple analytes and their ratios [27,28]. The application of second-tier testing before the release of the newborn screening result could reduce referrals and improve the positive predictive value for SCADD [8].

Twenty-one neonates with SCADD in this study underwent targeted sequencing of the ACADS gene using NGS. Sanger sequencing was used to validate suspected variants detected in neonates and their parents. All 21 cases received a definitive genetic diagnosis, leading to a 100% diagnostic rate. The pathogenic ACADS gene associated with SCADD is located on chromosome 12q24.31. It spans approximately 13 kb in length and encompasses 10 exons that encode a total of 412 amino acids [22]. Among the 21 confirmed neonates with SCADD, 15 cases exhibited compound heterozygous mutations, while 6 cases displayed homozygous mutations. The mutations were observed across multiple exons and introns, predominantly consisting of missense mutations. Notably, 18 neonates harbored pathogenic or likely pathogenic mutations. Four mutations of the ACADS gene detected in a pair of female twins and one male newborn with SCADD were assessed as variants of unknown clinical significance according to ACMG guidelines. However, in this study, they were observed to be pathogenic variants when present as compound heterozygous mutations. Among the 42 ACADS gene mutations identified in this study, the most prevalent mutation was c.1031A>G, accounting for 21.43% (9/42), followed by c.1130C>T, which accounted for 16.67% (7/42). According to the studies conducted by Gong et al. [7], the most common mutations of the ACADS gene in Beijing are c.1031A>G (3/11) and c.625G>A (3/11). In contrast, Zhao et al. [4] found that the most common mutations in Fujian is c.275C>G (9/16). In Zhejiang, the prevalent mutations are c.1031A>G (35.3%) and c.164C>T (20.6%) [12]; in Shandong, the common mutations are c.1031A>G and c.164C>T [5]; and c.1031A>G (11/21) in Changsha [9]. The research results in China consistently show that c.1031A>G is the most common mutation of the ACADS gene in the Chinese population.

However, the long-term consequences and the need for therapy in newborns with SCADD identified through NBS remain controversial. Many individuals with ACADS variations do not exhibit clinical symptoms; a primary issue is the lack of association between genotype and clinical phenotype, which raises the question of whether SCADD actually exists as a disease. Therefore, some NBS programs have removed SCADD from their screening panels [29]. As evidence accumulates that SCADD represents enzyme deficiencies with biochemical phenotypes rather than metabolic disorders with clinical implications, its inclusion in NBS panels should be evaluated according to local procedures. As part of the routine evaluation of procedures, both NBS programs and clinical genetics practices should assess SCADD management and develop a coordinated strategy that considers the impact of all system changes. On the other hand, the false-positive results due to ACADS gene variants may impose a burden on follow-up efforts, cause unnecessary anxiety for families, and lead to unnecessary interventions. Parents reported extreme anxiety during the diagnostic period and current feelings of uncertainty about their child’s future [5]. Because of insufficient research and a lack of long-term follow-up studies, there are no comprehensively accepted recommendations for appropriate therapy for patients with SCAD deficiency and symptoms. Clinicians should educate parents about the benign nature of these diagnoses and release them from follow-up without treatment. Current reports indicate that the incidence of SCADD in China remains relatively high, with some affected children exhibiting severe symptoms. The implementation of NBS in China has simplified the process and reduced associated costs. Therefore, it is worthwhile to screen newborns for SCADD in China.

In summary, our results suggest that NGS technology may be useful for confirming the diagnosis and assisting in clinical decision-making, our study also provides evidence indicating that SCADD diagnosed through NBS is primarily a benign condition, and early diagnosis is not necessarily essential. Currently, there is no consensus on whether SCADD should be classified as a disease; physicians should counsel families about potential risks. The continuous accumulation of data on newborns with SCADD, along with long-term follow-up and research on related pathogenesis, is essential for enhancing our understanding of SCADD.

## Figures and Tables

**Table 1:** The results of neonatal SCADD screening in Hefei from 2016 to 2023.

Year	Number of Live Births	Number of NBSs	Screening Rate (%)	Number of Suspected Cases	Number of Recall Suspected Cases	Number of Recall Positive Cases	Number of SCADD	Number of False-Positive Cases	False-Positive Rate (%)
2016	94,311	78,963	83.73	32	31	6	1	31	0.04
2017	136,528	130,940	95.91	55	52	9	2	53	0.04
2018	120,156	115,881	96.44	56	53	12	6	50	0.04
2019	118,594	116,657	98.37	45	43	7	3	42	0.04
2020	103,799	102,159	98.42	35	34	8	2	33	0.03
2021	87,784	87,239	99.38	33	32	4	1	32	0.04
2022	77,333	76,248	98.60	38	38	6	4	34	0.04
2023	75,517	74,843	99.11	28	28	5	2	26	0.03
Total	814,022	782,930	96.18	322	311	57	21	301	0.04

**Table 2:** Biochemical and genetic characteristics of newborns with SCADD.

No.	Gender	GA (w)	Birth Weight (g)	C4 (μmol/L)		Paternal		Maternal	
				Initial Screening	Secondary Screening	Locations	Variants DNA (Protein)	Locations	Variants DNA (Protein)
1	female	39 + 6	2600	1.29	2.69	exon5	c.578C>T (p.S193L)	exon9	c.1031A>G (p.E344G)
2	female	38 + 3	3500	1.20	1.23	exon8	c.989G>A (p.R330H)	exon8	c.989G>A (p.R330H)
3	female	36 + 0	2300	1.37	1.07	exon2	c.203C>G (p.A68V)	exon4	c.373G>C (p.G125R)
4	female	36 + 0	2000	1.63	1.03	exon2	c.203C>G (p.A68V)	exon4	c.373G>C (p.G125R)
5	female	37 + 0	3300	1.25	0.99	exon8	c.991G>A (p.A331T)	exon9	c.1031A>G (p.E344G)
6	female	39 + 4	3030	1.45	1.14	exon10	c.1157G>A (p.R386H)	exon9	c.1031A>G (p.E344G)
7	male	39 + 6	3400	1.29	1.04	exon10	c.1130C>T (p.P377L)	exon10	c.1130C>T (p.P377L)
8	male	41 + 3	3800	1.28	1.47	exon9	c.1031A>G (p.E344G)	exon10	c.1156C>T (p.R386C)
9	female	37 + 5	3450	1.99	1.82	exon2	c.164C>T (p.P55L)	exon10	c.1192C>T (p.Q398 *)
10	male	33 + 4	1600	1.32	2.09	exon9	c.1066G>A (p.A356T)	exon7	c.815G>A (p.R272H)
11	male	39 + 0	3500	2.07	1.75	exon10	c.1192C>T (p.Q398 *)	exon10	c.1192C>T (p.Q398 *)
12	male	39 + 2	3300	1.08	0.75	exon10	c.1130C>T (p.P377L)	exon10	c.1130C>T (p.P377L)
13	male	38 + 6	3000	0.74	1.13	intron7	c.933+1G>A	exon9	c.1031A>G (p.E344G)
14	male	39 + 2	4150	1.41	1.38	exon2	c.164C>T (p.P55L)	exon5	c.570G>A (p.W190)
15	male	40 + 1	3740	1.01	1.14	exon9	c.1031A>G (p.E344G)	exon10	c.1130C>T (p.P377L)
16	female	39 + 4	3300	1.18	1.08	exon10	c.1130C>T (p.P377L)	exon10	c.1130C>T (p.P377L)
17	female	39 + 3	3545	1.23	1.56	exon3	c.242C>A (p.A81D)	exon9	c.1054G>A (p.A352T)
18	female	38 + 2	2700	1.95	3.12	intron6	c.795+1G>A	exon9	c.1031A>G (p.E344G)
19	male	39 + 3	3970	2.42	2.33	exon3	c.320G>A (p.R107H)	exon9	c.1031A>G (p.E344G)
20	female	38 + 6	3320	1.96	1.55	exon9	c.1054G>A (p.A352T)	exon9	c.1031A>G (p.E344G)
21	male	39 + 2	3800	0.71	0.87	intron5	c.624+1G>T	exon10	c.1210G>A (p.G404R)

GA: gestational age; *: termination codon.

## Data Availability

The datasets generated and analyzed during the current study are not publicly available due to participant privacy concerns but are available from the corresponding author upon reasonable request.

## Abbreviations

ACADS	acyl-CoA dehydrogenase gene
C2	acetylcarnitine
C3	propionyl carnitine
C4	butyrylcarnitine
C5	isovalerylcarnitine
C6	hexanoylcarnitine
DBS	dried blood spot
IBDD	isobutyryl-CoA dehydrogenase deficiency
MADD	multiple acyl-CoA dehydrogenase deficiency
NBS	newborn screening
NGS	next-generation sequencing
SCADD	short-chain acyl-CoA dehydrogenase deficiency
